# Early Life Stress and the Onset of Obesity: Proof of MicroRNAs’ Involvement Through Modulation of Serotonin and Dopamine Systems’ Homeostasis

**DOI:** 10.3389/fphys.2020.00925

**Published:** 2020-07-28

**Authors:** Gabriel Araujo Tavares, Amada Torres, Julliet Araujo de Souza

**Affiliations:** ^1^Nantes Université, INRAE, UMR 1280, PhAN, Nantes, France; ^2^Laboratory of Neuroplasticity and Behavior, Graduate Program of Nutrition, Federal University of Pernambuco, Recife, Brazil; ^3^Developmental Genetics and Molecular Physiology, Instituto de Biotecnologia, Universidad Nacional Autonoma de Mexico – Campus Morelos, Cuernavaca, Mexico; ^4^Laboratory of Neuroplasticity and Behavior, Graduate Program of Neuropsychiatry and Behavioral Sciences, Federal University of Pernambuco, Recife, Brazil

**Keywords:** miRNA, early life stress, obesity, serotonin, dopamine

## Abstract

Healthy persons hold a very complex system for controlling energy homeostasis. The system functions on the interconnected way between the nutritional, endocrine, neural, and epigenetic regulation, which includes the microRNAs (miRNAs). Currently, it is well accepted that experiences of early life stress (ELS) carry modification of the central control of feeding behavior, one of the factors controlling energy homeostasis. Recently, studies give us a clue on the modulation of eating behavior, which is one of the main factors associated with the development of obesity. This clue connected the neural control through the serotonin (5HT) and dopamine (DA) systems with the fine regulation of miRNAs. The first pieces of evidence highlight the presence of the miR-16 in the regulation of the serotonin transporter (SERT) as well as the receptors 1a (5HT1A) and 2a (5HT2A). On the other hand, miR-504 is related to the dopamine receptor D2 (DRD2). As our knowledge advance, we expected to discover other important pathways for the regulation of the energy homeostasis. As both neurotransmission systems and miRNAs seem to be sensible to ELS, the aim of this review is to bring new insight about the involvement of miRNAs with a central role in the control of eating behavior focusing on the influences of ELS and regulation of neurotransmission systems.

## Introduction

Almost over one-third of the world’s population is overweight or obese (Chooi et al., 2019). This condition negatively affects the life quality, productivity, and costs with public health. One of the main aspects of this body weight regulation is feeding behavior (Remmers and Delemarre-van de Waal, 2011) which involves neural networks such as the serotonergic (5HT) and dopaminergic (DA) systems (Meguid et al., 2000). Several studies show that the disruption of those systems is strongly associated with increased food intake and/or preference for palatable food, which are important factors contributing for the onset of obesity (van Galen et al., 2018). Gene expression of both 5HT and DA systems can be influenced by miRNAs (Launay et al., 2011; Shi et al., 2014), and in this case, they would be the key regulatory molecules in the comprehension of the pathophysiology of the feeding behavior.

MiRNAs are small non-coding RNAs with an average length of approximately 22 nucleotides (Bartel, 2004). They regulate post-transcriptional gene expression by binding to the 3′UTR of mRNAs, some miRNAs also regulate the expression of another or several other miRNAs (Truscott et al., 2016), and even themselves (Zisoulis et al., 2012). Generally, miRNA specifically inhibit protein synthesis either by repressing translation or by inducing deadenylation and degradation of target mRNA (Bartel, 2004) but were also reported to activate translation (Huntzinger and Izaurralde, 2011). Each miRNA has the capacity to target hundreds of diverse transcripts, and a single messenger can be modulated by several miRNAs, this represents a highly coordinated system and fine-tuned regulation of protein expression (Krol et al., 2010; O’Carroll and Schaefer, 2013).

On the other hand, a healthy environment during the beginning of life is crucial for a proper development in mammals (Resnick et al., 1979; Morgane et al., 1993 2002). Maternal nutritional and emotional factors are critical during periconceptional and perinatal periods (Morgane et al., 1993; Chen and Baram, 2016). Early life stress (ELS) experiences can lead to long-term neurobehavioral complications. Both pre-clinical and clinical studies identify the influence of ELS on the development of several psychiatric disorders, including perturbation of feeding behavior, eating disorders and obesity (Chen and Baram, 2016; Entringer et al., 2016). Interestingly, the miRNAs are also sensible to ELS through several models, as showed on Table 1. In this context, this review brings a potential role of the miRNAs in the onset of obesity through modulation of 5HT and DA in response to ELS.

**TABLE 1:** Influences of ELS on miRNA activity.

**ELS model**	**Subjects**	**Region**	**Outcome**	**Authors**
**Pre-clinical studies**				
MS	Rat	Hippocampus	Increased miR-16	Bai et al., 2012
MS	Mice	Cortical neurons	Impaired response of miR-212 to the learning process on a cocaine conditioned place preference test	Viola et al., 2016
MS + CUS	Rat	NAcc	Increased miR-504	Zhang et al., 2013
MS	Rat	mPFC	Increased REST4	Uchida et al., 2010
Morphine + Apnea + MS	Mice	Hippocampus	Decreased miR-204-5p, miR-455-3p, miR-448-5p, and miR-574-3p	McAdams et al., 2015
CUMS	Rat	Basolateral amygdala	Increased rno-miR-124a	Xu et al., 2017
Increased maternal care	Rat	Hypothalamus	increased rno-miR-488, rno-miR-144, and rno-miR-542-5p and decreased rno-miR-421 and rno-miR-376b-5p	Vogel Ciernia et al., 2018
Protein malnutrition	Mice	Hypothalamus	increased mmu-miR-187-3p, mmu-miR-369-3p and mmu-miR-132-3p	Berardino et al., 2019
Unpredictable maternal separation combined with maternal stress	Mice	Sperm	Changes in miRNA transmitted to F2 generation	Gapp et al., 2014
Prenatal stress	Rat	Hippocampus	Decreased hsa-miR-125b-1-3p	Cattane et al., 2019
**Clinical studies**				
Childhood maltreatment	both sexes	Leukocytes	Methylation changes in CpGs close to region coding miR-124-3	Prados et al., 2015
Childhood abuse	Men aged 45 years old	Whole blood	Methylation changes in promoter region of 39 miRNAs	Suderman et al., 2014
Child abuse	European adults of both sexes	Buccal mucosa cells	Association between the polymorphism rs3125 of 5HT2A and brooding. This region is predicted to be targeted by miR-1270, miR-1304, miR-202, miR-539 and miR-620	Eszlari et al., 2019
Childhood trauma	Adult of both sexes	Blood cells	Decreased hsa-miR-125b-1-3p	Cattane et al., 2019
Childhood trauma	Adult both sexes	Human hippocampus progenitor cells	Decreased hsa-miR-125b-1-3p	Cattane et al., 2019

*ELS, early life stress; MS, maternal separation; CUS, chronic unpredictable stress; CUMS, chronic unpredictable mild-life stress; NAc, nucleus accumbens; mPFC, medial pre-frontal cortex; CpGs, methylated cytosines followed by guanine nucleotide sites.*

## Serotonin: Role on Feeding Behavior, Influences of ELS, and miRNA Regulation

The 5HT system includes receptors, transporters and enzymes involved in the metabolism of serotonin (5-Hydroxytryptamine), and it regulates several functions in the organism as locomotors activity, body temperature, wake-sleep cycle, and feeding behavior (Lam et al., 2010; Olivier, 2015). Regarding the control of eating behavior, serotonin has a well-established anorectic role through promotion of satiety. In the arcuate nucleus of the hypothalamus, serotonin acts in different ways; it acts on 5HT1B, promoting inhibition of neurons that produce neuropeptide Y (NPY) and the cocaine and amphetamine-related transcript (CART), which are orexigenic. It also acts on 5HT2C, promoting activation of neurons that produce pro-opiomelanocortin (POMC) and the peptide related to the agouti gene (AgRP), which are anorexigenic, thus promoting satiety signaling (Heisler et al., 2006). In addition, recent studies also refer that serotonin has a role in the hedonic regulation of eating behavior. Receptors such as 5HT6 in areas of the mesocorticolimbic circuit have been associated with motivational feeding behavior (da Silva et al., 2017). The impairments of the homeostasis of the serotonergic system are associated with disorders of eating behavior, usually associated with increased food intake, either by homeostatic or hedonic changes. In particular, the serotonergic system appears extremely sensitive to environmental changes during the development of the organism, and several studies have shown that ELS impairs the function of the 5HT system (de Lima et al., 2020).

Models of ELS in animals are usually associated with deprivation of the mother–infant relationship, such as in maternal separation (MS) and early weaning (EW) models (Kikusui and Mori, 2009; Harrison and Baune, 2014). Previous studies from our laboratory show that the MS disrupts the 5HT system. In middle aged females, it increases the 5HT1B gene expression in the hypothalamus, associated with decreased food intake (de Souza et al., 2020a), and in adult males, we observed a decreased action of fluoxetine on food intake (de Souza et al., 2020b). In addition, MS promotes decreased 5HT concentration in hypothalamus and amygdala of young animals, associated with increased palatable food intake (de Lima et al., 2020). Together, these data suggest that MS alter the serotonergic system function, contributing to disorders of feeding behavior. On the other hand, we have been able to associate the EW with changes in gene expression of several components of the 5HT system in male and female rats, such as SERT, 5HT1B, and 5HT2C in hypothalamus and brainstem. Based on the patterns of expression in the brainstem and response to fenfluramine, we suggested a hypofunction of the serotonergic system in the EW animals (Tavares et al., 2019, 2020a,b). All these changes in the 5-HT system were accompanied by alterations on feeding behavior, which indicate that the 5HT system control of feeding behavior can be modulated by ELS, which can be directly linked to the onset of obesity.

Recently, studies have deepened about these compensatory changes and epigenetic modifications have been extensively investigated. In this respect, miRNAs have been shown to be important regulators y/o mediators of gene expression. In the case of depression, it is currently accepted that several miRNAs modulate the activity of the serotonergic system, but little is known about these regulators in the context of eating behavior. As far as we know, miR-16 is able to bind the SERT messenger (Table 2) and silence its expression in humans and animals (Baudry et al., 2010; Moya et al., 2013; Song et al., 2015; Shao et al., 2018). The relationship between miR-16 and SERT is even modulated by pharmacological antidepressant treatment and also alternative treatments as the electroacupuncture; besides different responses according to the affected brain area, these treatments improve the level of depressive behaviors, suggesting a highly specific regulation (Baudry et al., 2010; Zhao et al., 2019). SERT appears to be a key piece of regulation, as different miRNAs can modify its expression, as the mmu-miR-135 (Issler et al., 2014), rno-miR-18a-5p, rno-miR-34a-5p, rno-miR-135a-5p, rno-miR-195-5p, rno-miR-320-3p, rno-miR-674-3p, and rno-miR-872-5p (Zurawek et al., 2017). This relationship between miR-16 and SERT is interesting, since SERT activity is directly related to serotonergic signaling. SERT recaptures the remaining amount of serotonin from the synaptic clefts, and an increase in its activity may mean a decrease in serotonergic signaling. In depression, has been shown that decreased levels of miR-16 and elevated levels of SERT are associated with the pathology by promoting a reduction in serotonergic signaling. Drugs that block SERT activity and increase serotonin levels are used to treat this depressive behavior. Interestingly, the same drugs are used to treat obesity (Halford et al., 2012) as they also promote a reduction in food intake. This evidence gives a primary role to miR-16 that may also be a candidate to modulate SERT activity in the context of eating disorders.

**TABLE 2:** Components of the serotonergic and dopaminergic systems and their regulatory-associated miRNAs.

**Components**	**miRNAs**	**Authors**
**Serotonin or 5-Hydroxytryptamine (5HT) system**		
SERT (SLC6A4/5HTT)	miR-16, miR-135, miR-18a-5p, miR-34a-5p, miR-135a-5p, miR-195-5p, miR-320-3p, miR-674-3p, and miR-872-5p.	Baudry et al., 2010; Launay et al., 2011; Moya et al., 2013; Issler et al., 2014; Song et al., 2015; Zurawek et al., 2017; Shao et al., 2018; Zhao et al., 2019
5HT1A	miR-16, miR-135, and miR-26a-2.	Liu et al., 2017; Xie et al., 2019
5HT1B	miR-96	Jensen et al., 2009
5HT2A	miR-16	Yang et al., 2017
5HT2C	miR-34	Andolina et al., 2016
5HT4	miR-103, miR-15b, and a mix containing miR-103, miR-15b, and miR-16	Wohlfarth et al., 2017
5HT7	miR-29a	Volpicelli et al., 2019
**Dopamine (DA) system**		
DRD1	miR-504, miR-105, miR-15a, miR-15b, miR-16 and miR-142-3p	Tobón et al., 2012, 2015; Zhang et al., 2013; Zhao et al., 2017; Wu et al., 2020
DRD2	miR-143, miR-200a, miR-504, has-miR-9 and miR-326	Zhang et al., 2013, 2015; Shi et al., 2014; Gangisetty et al., 2017; Wu et al., 2018; Mavrikaki et al., 2019; Wang et al., 2019
DRD3	let-7d	Bahi and Dreyer, 2018
DAT	miR-137 and miR-491	Jia et al., 2016

*SERT, solute carrier family 6 member 4 (SLC6A4/5HTT), serotonin transporter; 5HT1A, 5-Hydroxytryptamine receptor 1A; 5HT1B, 5-Hydroxytryptamine receptor 1B; 5HT2C, 5-Hydroxytryptamine receptor 2C; 5HT4, 5-Hydroxytryptamine receptor 4; 5HT7, 5-Hydroxytryptamine receptor 7; DRD1, dopamine receptor D1; DRD2, dopamine receptor D2; DRD3, dopamine receptor D3; DAT, dopamine transporter, solute carrier family 6 member 3 (SLC6A3).*

In addition to SERT, miRNAs modulate the activity of other components of the serotonergic system (Table 2), such as 5HT1B, 5HT1A, 5HT4, 5HT2C, and 5HT7. The 5HT1B receptor is advised as a target of the miR-96 (Jensen et al., 2009). 5HT1A seems to be targeted by miR-16, miR-135 (Liu et al., 2017), and has-miR-26a-2 (Xie et al., 2019). The 5HT4 receptor acquire decreased expression in response to miR-103, has-miR-15b and a mix containing hsa-miR-103, has-miR-15b and hsa-miR-16 (Wohlfarth et al., 2017). In addition, miR-34 appears to bind the receptor 5HT2C (Andolina et al., 2016), hsa-miR-16 appears to reduce 5HT2A expression (Yang et al., 2017), and miR-29a decreases the expression of 5HT7 (Volpicelli et al., 2019). The impairment of the activity of these receptors is associated with disrupted food intake either by homeostatic or hedonic mechanisms. 5HT1A, 5HT1B, and 5HT2C are strongly associated with satiety signaling, and several studies report that their disruption promotes increased food intake. On the other hand, 5HT4 is associated with hedonic modulation of food intake and obesity. Thus, the modulation of these receptors through miRNAs can also be associated with the onset of eating disorders leading to obesity.

## Dopamine: Role on Feeding Behavior, Influences of ELS, and miRNA Regulation

The dopaminergic system, as well as the serotoninergic system, comprises a set of neurotransmitter, enzymes, receptors, and dopamine transporter (DAT). On the other hand, neurons that synthesize dopamine can be found in the brainstem and can be divided into three groups, which forms the Nigro Striatal system, the mesocorticolimbic system, and the mesocortical system (Ogawa and Watabe-Uchida, 2018). The principal role on feeding behavior is taken by the mesocorticolimbic system (Wise, 1989; Berridge and Kringelbach, 2008). Dopaminergic neurons are known to be involved in emotion-based behavior including motivation and reward (Phillips et al., 2008). Therefore, in the context of the feeding behavior, this system is mainly related to the hedonic component of feeding, but evidences also point out that dopamine is a key component on hypothalamic regulation of the homeostatic eating behavior (Meguid et al., 2000; Ikeda et al., 2018).

The DA system is sensible to ELS and its disruption is associated with several psychiatric disorders, such as eating disorders and obesity (Naef et al., 2015). Our previous study showed that DRD1 and DRD2 gene expression were increased in the brainstem of adult rats, accompanied by higher palatable food intake after MS (de Souza et al., 2018). The MS also modulates the DA system in other brain areas, such as PLC, NAcc, and striatum, changing the density of immunoreactive fibers of TH, and the mRNA expression of DRD2, DRD1, and DRD5 (Majcher-Maślanka et al., 2017). On the other hand, EW increases DRD1 mRNA expression in the hypothalamus and brainstem and DRD2 in the brainstem of middle-aged male rats (Tavares et al., 2020b). In all of these studies, disrupted patterns on feeding behavior are observed, indicating that alterations in the dopaminergic system can be one of the underlying mechanisms that lead to behavioral disorders.

Increased evidence points out that several components of the dopaminergic system are influenced by some miRNAs (Table 2). DRD1 appears to be regulated by miR-504 (Zhang et al., 2013), rno-miR-105 (Zhao et al., 2017), and for the cluster of hsa-miR-15a-5p, hsa-miR-15b-5p, and hsa-miR-16-5p, and mmu-miR-142-3p (Tobón et al., 2012, 2015). The expression of the DRD2 is modified by miR-143-3p (Wang et al., 2019), miR-200a (Wu et al., 2018), miR-504 (Zhang et al., 2013), hsa-miR-9, and hsa-miR-326 (Shi et al., 2014; Zhang et al., 2015; Gangisetty et al., 2017; Mavrikaki et al., 2019). Both receptors, DRD1 and DRD2, are associated with control of food intake, either homeostatic or hedonic, in several areas of the brain (Wise, 1989; Ikeda et al., 2018) which indicates that its modulation through miRNAs can modulate the food intake. In addition, overexpression of let-7d is negatively correlated with the expression of DRD3 in the hippocampus of mice (Bahi and Dreyer, 2018). The activity of the DRD3 is controversy in the context of food intake, but some evidences associate it with eating disorders and decreased food intake (Thomsen et al., 2017; González et al., 2019). The expression of DAT, the major controller of dopamine levels in the synaptic clefts, is post-transcriptionally regulated on cell culture of dopaminergic neurons by miR-137 and miR-491 (Jia et al., 2016). This transporter acts like SERT, reuptaking the dopamine from the synaptic cleft, so its function is extremely necessary to normal DA signalization, even in the context of eating behavior. On the other hand, the reduction of Dicer, a miRNA-processing ribonuclease III, in the ventral midbrain of DA neurons promotes changes in the miRNAs profile and altered the survival capacity of these dopaminergic neurons (Chmielarz et al., 2017). Together, these evidences extended the susceptibility of the DA system to the regulation of miRNAs, which can lead to modulation of eating behavior and may be associated with eating disorders.

## Perspectives: Role of the miRNAs on the Onset of Obesity Through 5HT and DA Systems’ Disruption in the Context of ELS

In addition to knowing that components of the 5HT and DA neurotransmission systems are susceptible to ELS, some evidence also shows that miRNAs have their expression and activity influenced by ELS, which is summarized in Table 1. Both, pre-clinical and clinical studies affirm that childhood trauma could be associated with the modulation of miRNA, as the case of the miR-16 and miR-504 which have their control of the serotonin and dopamine impaired by stress, with consequences such as depression, anhedonia, and body weight gain. However, more studies are needed to understand the full picture, specifically in the context of the control of the feeding behavior, which is directly involved in the development of obesity.

Conversely, both clinical and pre-clinical studies demonstrate that ELS is able to alter SERT activity (Wankerl et al., 2014; Van Der Knaap et al., 2015; Tavares et al., 2019, 2020a). Interestingly, differences in SERT activity are observed in obesity, both in humans and animals (Giannaccini et al., 2013; Borgers et al., 2014; Zha et al., 2017). For example, the density of SERT is reduced in obese humans (Giannaccini et al., 2013; Borgers et al., 2014) and increased in rats with abdominal obesity who were exposed to a diet rich in simple carbohydrates (Spadaro et al., 2015). In addition to being involved in the pathophysiology of obesity and being sensitive to ELS, several lines of evidence in the literature show that SERT is a target for miR-16 and propose an important role in regulating its activity (Baudry et al., 2010). On the other hand, animal studies demonstrate that the 5HT1A receptor is also modulated by ELS (Bravo et al., 2014; Razoux et al., 2017) and has increased density in the hippocampus and hypothalamus of rats chronically submitted to a Westernized diet (Yu et al., 2018). Interestingly, 5HT1A is also the target of miR-16, which has its expression modulated by ELS (Bai et al., 2012). The receptor 5HT2A is, as well, modulated by ELS in animals and humans (Rentesi et al., 2013; Parade et al., 2017) and involved with the pathophysiology of obesity (Rosmond et al., 2002; Huang et al., 2004). Interestingly, the 5HT2A is also targeted by the miR-16 (Yang et al., 2017). From these observations, we believe that miR-16 is an excellent candidate for moderating changes in SERT, 5HT1A, and 5HT2A due to ELS, in the context of the altered eating behavior.

Regarding the dopaminergic system, the DRD1 and DRD2 actively participate in the regulation of food intake, especially with regard to palatable foods, as these are related to the food reward system (Meguid et al., 2000; Berridge et al., 2009; Volkow et al., 2011). Changes in this reward system are linked to eating behavior disorders, with changes in the activity of DRD1 and DRD2 being observed in humans and animals (Guo et al., 2014; Rivera et al., 2015; Gaiser et al., 2016; de Souza et al., 2018; Romanova et al., 2018; Tavares et al., 2020b). In addition, both receptors are modulated by ELS (de Souza et al., 2018; Tavares et al., 2020b). Interestingly, we observed that miR-504 targets both DRD1 and DRD2, with their expression being altered by ELS (Zhang et al., 2015). Additionally, DRD1 has also been identified as a target for miR-16 (Wu et al., 2020). Thus, we believe that miR-504 and miR-16 modulate DRD1 and DRD2, in the context of eating disorders associated with ELS.

In summary, according to the evidence reported, we can infer that the serotonergic and dopaminergic systems undergo regulation of their activity through post-transcriptional modulation by miRNAs. Both systems participate in the physiological and pathological processes of eating behavior, which leads us to believe that miRNAs may be behind several changes in eating behavior as observed in several disorders such as obesity. Several studies point out that the genesis of these disorders is largely associated with experiences of stress early in life. Neonatal stress is already well described as a modulator of the serotonergic and dopaminergic systems associated with disorders of eating behavior, as well as a modulator of expression and activity of miRNAs. In addition, we know that miRNAs participate in the pathological processes of several psychiatric disorders. Thus, we establish here a relationship between neonatal stress and the modulation of the serotonergic and dopaminergic systems, through post-transcriptional regulation by miRNAs, as a possible pathophysiological mechanism behind eating behavior disorders. Future studies are needed to investigate this relationship and provide further support for the scientific community in the search for understanding and treatment of pathologies of eating behavior.

## Author Contributions

All authors listed have made a substantial, direct and intellectual contribution to the work, and approved it for publication.

## Conflict of Interest

The authors declare that the research was conducted in the absence of any commercial or financial relationships that could be construed as a potential conflict of interest.

## Abbreviations

3′UTR: 3′Untranslated region
5HT: 5-Hydroxytryptamine, serotonin system
5HT1A: 5-Hydroxytryptamine receptor 1A
5HT1B: 5-Hydroxytryptamine receptor 1B
5HT2C: 5-Hydroxytryptamine receptor 2C
5HT4: 5-Hydroxytryptamine receptor 4
5HT6: 5-Hydroxytryptamine receptor 6
5HT7: 5-Hydroxytryptamine receptor 7
AgRP: protein related to gene agouti
cAMP: cyclic adenosine monophosphate
CART: cocaine and amphetamine-related transcript
CpGs: methylated cytosines follow of guanine nucleotide sites
CUMS: chronic unpredictable mild-life stress
CUS: chronic unpredictable stress
DA: dopamine, dopamine system
DAT: solute carrier family 6, neurotransmitter transporter dopamine member 3, SLC6A3
Dicer: microRNA-processing ribonuclease III
DRD1: dopamine receptor D1
DRD2: dopamine receptor D2
DRD3: dopamine receptor D3
DRD5: dopamine receptor D5
ELS: early life stress
EW: early weaning
has-mir-16: MI0000070, MI0000115
Let-7d: hsa-let-7d-5p, mmu-let-7d-5p, rno-let-7d-5p (MIMAT0000065, MIMAT0000383, MIMAT0000562)
miR-103: has-mir-103a-1 MI0000109, has-mir-103a-2 MI0000108
miR-143-3p: hsa-miR-143-3p, mmu-miR-143-3p, rno-miR-143-3p (MIMAT0000435, MIMAT0000247, MIMAT0000849)
miR-16-5p: hsa-miR-16-5p, mmu-miR-16-5p, rno-miR-16-5p (MIMAT0000069, MIMAT0000527, MIMAT0000785)
miR-200a: rno-miR-200a-3p, mmu-miR-200a-3p, hsa-miR-200a-3p (MIMAT0000682, MIMAT0000519, MIMAT0000874)
miR-96: hsa-miR-96-5p, mmu-miR-96-5p, rno-miR-96-5p (MIMAT0000095, MIMAT0000541, MIMAT0000818)
miRNAs: microRNAs
mmu-miR-135: MI0000161, MI0000715
mPFC: medial pre-frontal cortex
mRNA: Messenger RNA
MS: maternal separation
NAcc: nucleus accumbens
NPY: neuropeptide Y
PLC: prelimbic cortex
PND: postnatal day
POMC: pro-opiomelanocortin
REST: repressor element-1 silencing transcription factor
RNAs: ribonucleic acids
SERT: solute carrier family 6 member 4 (SLC6A4/5HTT), serotonin transporter
TH: tyrosine hydroxylase.
